# Anterolateral ligament injury in ACL‐injured patients is not associated with the presence of the deep lateral femoral notch sign: A cross‐sectional study

**DOI:** 10.1002/jeo2.70950

**Published:** 2026-10-09

**Authors:** David Santos‐Nunes, Daniel Medina‐Dias, Pedro Poças, Pedro Sá, Miguel Castro, Paulo Oliveira, Francisco Serdoura, Manuel Gutierres

**Affiliations:** ^1^ Faculdade de Medicina da Universidade do Porto Porto Portugal; ^2^ Unidade Local de Saúde de São João, Serviço de Ortopedia e Traumatologi Porto Portugal; ^3^ Unidade Local de Saúde de São João, Serviço de Radiologia Porto Portugal

**Keywords:** anterior cruciate ligament, anterior tibial translation, anterolateral ligament, anterolateral rotatory instability, deep lateral femoral notch sign, magnetic resonance imaging

## Abstract

**Purpose:**

The anterolateral ligament (ALL) has been proposed as a secondary stabiliser of the knee against tibial internal rotation in anterior cruciate ligament (ACL)‐injured knees. The deep lateral femoral notch sign (DLFNS) has been proposed as an indirect imaging marker of high‐energy pivot‐shift trauma in ACL‐injured knees. This study investigates the association between ALL injury and magnetic resonance imaging (MRI) anterolateral rotatory instability signs—including DLFNS and medial and lateral anterior tibial translation—in acute ACL injuries.

**Methods:**

A cross‐sectional study was conducted on 176 patients (aged 18–50) who underwent primary ACL reconstruction. Patients were stratified by MRI‐based ALL status: injured and intact. Two observers independently assessed ALL injury. Primary outcomes were the presence of DLFNS (depth > 2 mm) and medial and lateral anterior tibial translation.

**Results:**

ALL injury was identified in 40/176 (22.7%) of patients with acute ACL injury. Interobserver agreement for ALL injury assessment was substantial (*κ* = 0.715). DLFNS prevalence did not differ significantly between ALL‐injured and intact groups (10/40 (25.0%) vs. 29/136 (21.3%); *p* = 0.623). No significant differences were found in medial or lateral anterior tibial translation between groups. On multivariable logistic regression, concomitant bone bruise was the only factor independently associated with ALL injury (adjusted odds ratio 2.192, 95% confidence interval [1.012–4.748]; *p* = 0.047), whereas no association was found with time to MRI or LFNS.

**Conclusions:**

No direct association was found between ALL injury and MRI signs of anterolateral rotatory instability, and bone bruise was the only independent factor associated with ALL injury. This finding may be consistent with ALL injury occurring within a broader injury pattern, which may not be reliably captured by DLFNS alone. The significantly greater lateral than medial anterior tibial subluxation suggests increased tibial internal rotation in ACL‐injured knees, independent of ALL injury status.

**Level of Evidence:**

Level III, cross‐sectional study.

## Graphical abstract

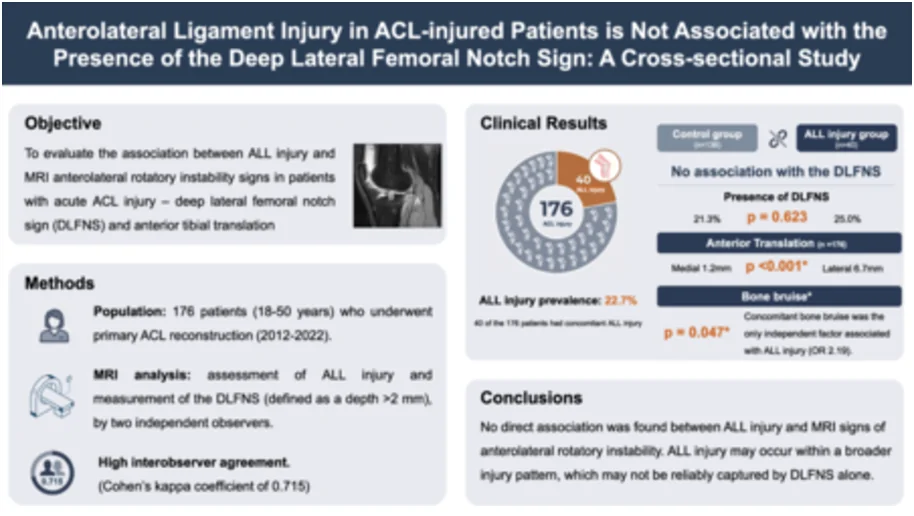

**Abbreviations**
ACL: anterior cruciate ligament
ALC: anterolateral complex of the knee
ALL: anterolateral ligament
BMI: body mass index
CI: confidence interval
DLFNS: deep lateral femoral notch sign
ICD: International Classification of Diseases
IQR: interquartile range
LCL: lateral collateral ligament
LFNS: lateral femoral notch sign
LPTS: lateral posterior tibial slope
MBA: meniscal bone angle
MCL: medial collateral ligament
MRI: magnetic resonance imaging
PCL: posterior cruciate ligament

## INTRODUCTION

Anterior cruciate ligament (ACL) injuries are among the most common serious knee injuries in young, physically active individuals participating in pivoting sports, with an annual incidence of 68.6–80.0 per 100,000 person‐years [26, 46]. These injuries lead to significant functional limitations, lower return‐to‐sport rates, and an elevated risk of developing early post‐traumatic osteoarthritis, even after surgical reconstruction [6, 26, 36]. Residual rotational instability, most commonly assessed by the pivot‐shift test, is a key determinant of clinical outcomes and graft survival [2, 40]. Persistent high‐grade pivot shift is associated with inferior patient‐reported outcomes and higher re‐rupture rates, highlighting the limitations of isolated ACL reconstruction in fully restoring rotational knee stability [40, 45].

The anterolateral complex (ALC), which includes the anterolateral ligament (ALL), is an important secondary stabiliser that contributes to the control of tibial internal rotation and prevents the pivot‐shift phenomenon [31, 50]. The reported prevalence of ALL injury in ACL‐injured knees varies widely, ranging from 11% to 79%, depending on imaging criteria [1]. This injury pattern has been associated with increased rotational laxity, higher‐grade pivot shift, and impaired dynamic stability [18, 31, 50]. These findings suggest that unrecognised or untreated ALL injury may be associated with residual rotational instability and graft overload following isolated ACL reconstruction. Consequently, ALL reconstruction or lateral extra‐articular tenodesis has been proposed in selected high‐risk patients to optimise rotational stability and reduce the risk of graft failure [12, 31, 39, 51].

The lateral femoral notch sign (LFNS) has been proposed as an indirect imaging marker of high‐energy pivoting trauma in ACL‐injured knees [5, 7, 13]. Dimitriou et al. demonstrated that a LFNS measured on plain radiographs exceeding 1.8 mm was associated with ALL injury in ACL‐injured patients [7]. In ACL‐injured knees, Braz et al. demonstrated that a deep lateral femoral notch sign (DLFNS) exceeding 2 mm is associated with a higher incidence of lateral meniscal injuries and a steeper lateral posterior tibial slope [4]. More recent studies have associated DLFNS with a broader injury pattern, including lateral meniscal tears, posterior root tears, and altered distal femoral morphology [19, 24, 38]. However, conflicting evidence exists regarding its specificity for concomitant ALL injury; notably, Kanakamedala et al. [27] reported no association between notch depth and rotatory instability, and Huang et al. [22] demonstrated that DLFNS failed to predict dynamic anterior tibial laxity. Although expert consensus supported anterolateral augmentation in selected high‐risk patients, such as those with a high‐grade pivot shift or participation in pivoting sports [20, 49], there are currently no standardised clinical guidelines that incorporate DLFNS as a decision‐making criterion [12, 20, 49].

The main aim of this study is to evaluate the association between ALL injury and magnetic resonance imaging (MRI) anterolateral rotatory instability signs—including DLFNS and medial and lateral anterior tibial translation—in patients with acute ACL injury. It was hypothesised that concomitant ALL injury would be associated with MRI signs of anterolateral rotatory instability, including DLFNS and increased medial and lateral anterior tibial translation.

## MATERIALS AND METHODS

This cross‐sectional study was performed in the authors' Hospital Department of Orthopaedics and Traumatology and followed the ethical guidelines of the Declaration of Helsinki. Patient inclusion and MRI data were retrospectively collected from January 2012 to December 2022. Data extraction, image analysis, and manuscript preparation were carried out between December 2025 and January 2026. Approval was obtained from the local hospital's Ethics Committee (CE‐425‐2025).

### Participants

The target population included patients aged 18–50 years who underwent primary ACL reconstruction for complete ACL injury between January 2012 and December 2022. Patients were selected consecutively from the hospital's electronic database, according to International Classification of Diseases (ICD)−9 and ICD‐10 coding. Inclusion criteria were: (1) adult patients aged 18–50 years at the time of surgery; (2) diagnosis of complete acute ACL injury confirmed on preoperative MRI; (3) underwent primary ACL reconstruction between January 2012 and December 2022. Exclusion criteria were: (1) previous ipsilateral knee surgery; (2) absence of preoperative MRI data; (3) MRI of insufficient quality to perform the defined measurements; (4) MRI acquired with a knee flexion angle of 20° or greater. Patients with complete ACL injuries were divided in two groups: (1) control group: patients with an intact ALL; (2) ALL group: patients with an ALL injury on MRI.

### Outcomes

The primary outcome of this study was to evaluate the association between ALL injury and MRI anterolateral rotatory instability signs—DLFNS and medial and lateral anterior tibial translation—in patients with complete acute ACL injury. Anterior tibial translation was included as a surrogate marker of anterolateral rotatory instability, as compartmental asymmetry between medial and lateral subluxation on static MRI has been proposed as an indirect indicator of tibial internal rotation in ACL‐injured knees [33, 59].

The secondary outcome was to assess the association of ALL injury and concomitant injuries—bone bruise (defined as increased signal intensity on T2‐weighted images of the lateral femoral condyle or lateral tibial plateau), medial and lateral meniscus, medial and lateral collateral ligaments (MCL and LCL, respectively), posterior cruciate ligament (PCL)—and knee morphological characteristics—lateral posterior tibial slope (LPTS), and meniscal bone angle (MBA).

### Data collection and MRI measurements

All patient data were extracted from electronic medical records. The Ethics Committee waived the requirement for informed consent due to its retrospective nature. The following variables were collected: age; sex; body mass index (BMI); injury mechanism; and time to MRI (defined as the interval between injury and MRI acquisition, expressed in days).

MRI measurements were performed using the Sectra UniView PACS® programme. MRI acquisition protocols remained standardised and consistent throughout the 10‐year study period. MRI scans were acquired using a 1.5 T system with a field of view of 160 mm. Sagittal 3 mm proton density‐weighted sequences with fat saturation, T1‐weighted sequences, and T2*‐weighted sequences were analysed. During image acquisition, patients were positioned supine with the knee in near extension (flexion < 20°), as confirmed using the method described by Harmes et al. [14]. MRI parameters included depth of LFNS (mm), ALL injury, medial and lateral anterior tibial translation, bone bruise, medial and lateral meniscal injuries, MCL and LCL injuries, PCL injuries, LPTS, and MBA. One observer (D.S.N.) performed all measurements except for ALL injury assessment. Two radiology residents (P.P. and P.S.) with a special interest in musculoskeletal pathology independently evaluated ALL injuries due to inherent identification challenges. Radiological data collection was supervised by a senior musculoskeletal radiologist (M.C.), who assisted in resolving interobserver discrepancies. Interobserver agreement for ALL injury was assessed using Cohen's kappa coefficient (*κ*). The LFNS was measured on the sagittal slice demonstrating the deepest impression of the notch, using the tangent method described by Warren et al. [55] (Figure 1).

**Figure 1:**
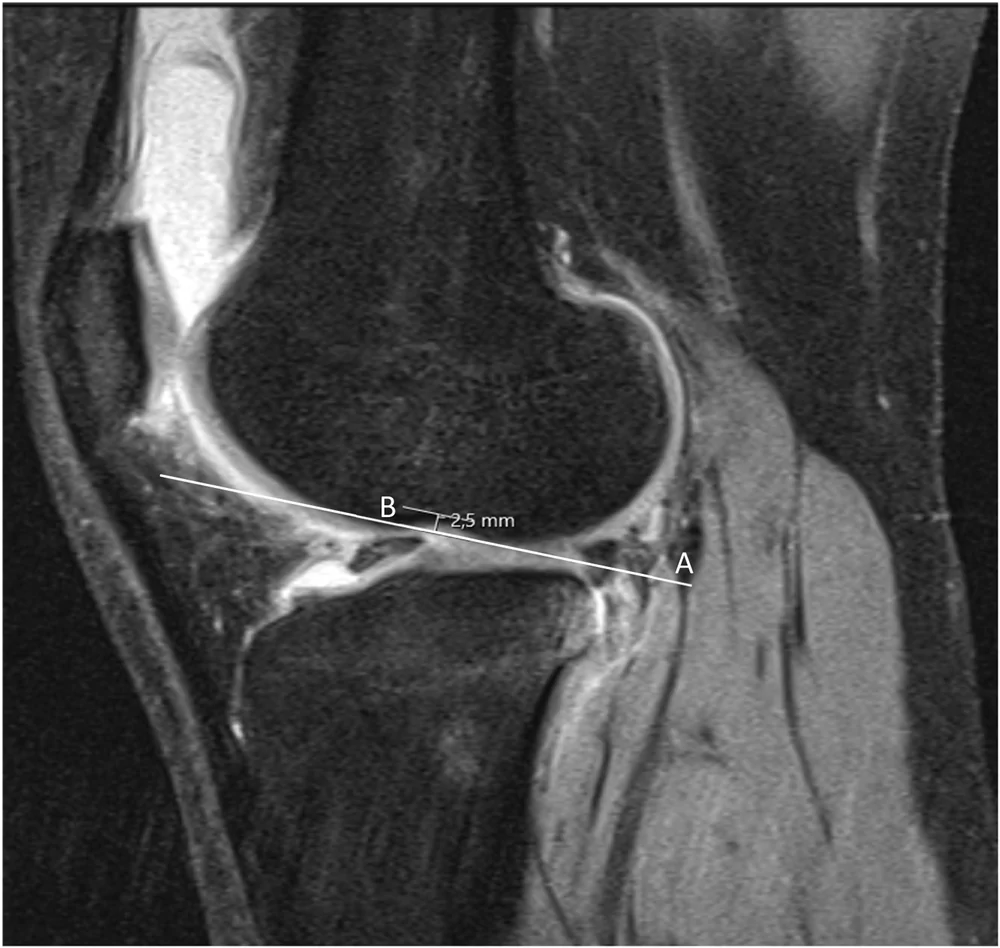
Measurement of the lateral femoral notch sign (LFNS) depth according to the method described by Warren et al. [55]. A tangent line (A) is drawn along the subchondral surface of the lateral femoral condyle. A perpendicular line (B) is drawn from the deepest point of the notch to the tangent line. The distance between these two points represents the notch depth in millimetres (mm). A depth exceeding 2 mm is classified as a positive deep lateral femoral notch sign (DLFNS). Sagittal T2*‐weighted magnetic resonance imaging.

LFNS was recorded as a continuous variable in millimetres (mm), and a cutoff value of >2 mm was used to define DLFNS positivity, in accordance with previous literature [19]. The ALL was assessed according to the method described by Muramatsu et al. [44] on coronal and axial fat‐suppressed proton density‐weighted sequences (Figure 2).

**Figure 2:**
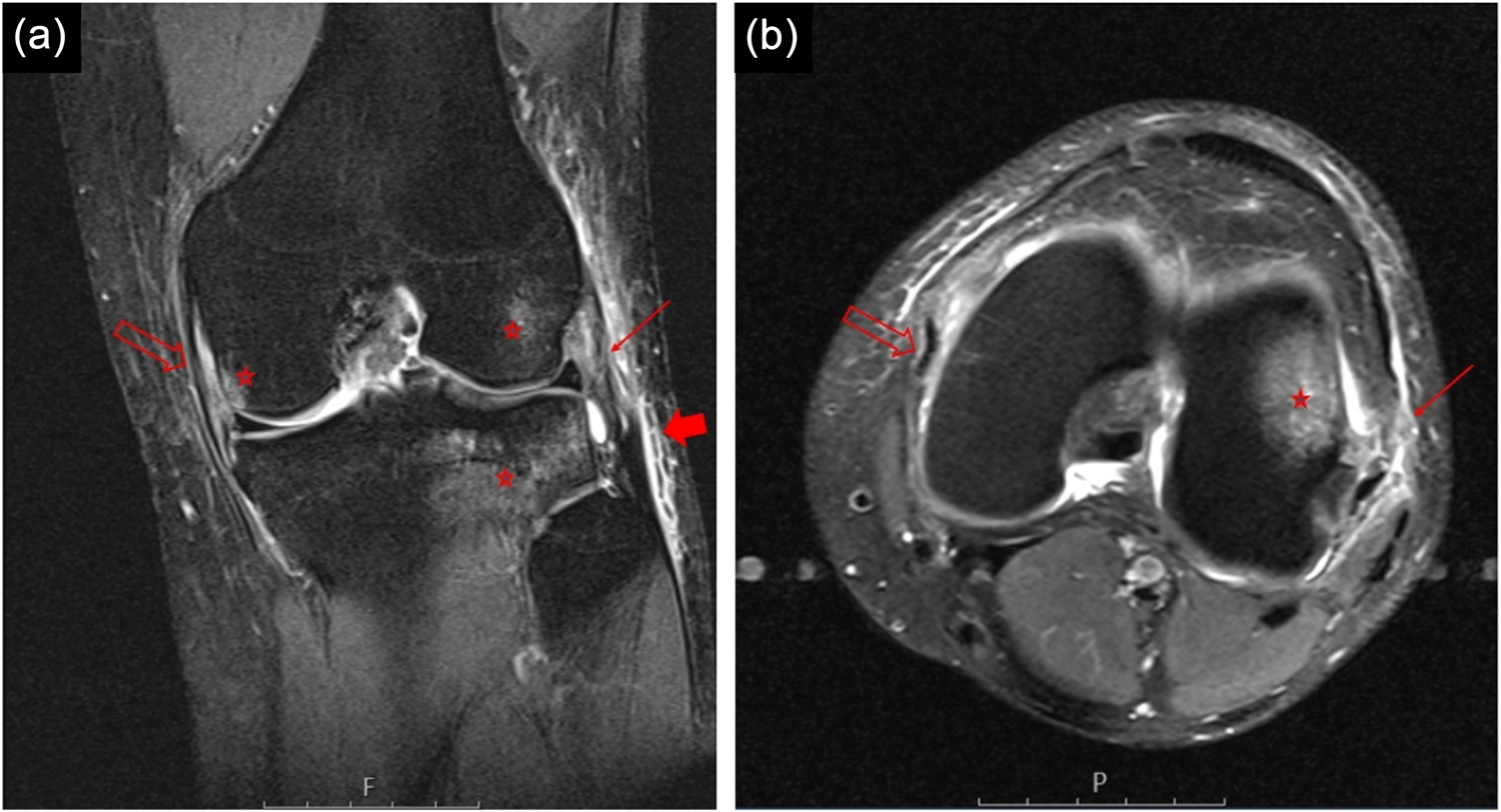
Assessment of the anterolateral ligament (ALL) on magnetic resonance imaging (MRI) in a patient with anterior cruciate ligament (ACL) injured, according to the method described by Muramatsu et al. [44]. (a) Coronal fat‐suppressed proton density‐weighted MRI. (b) Axial fat‐suppressed proton density‐weighted MRI. Thin arrow: ALL disruption; thick arrow: periarticular soft tissue oedema; empty arrow: medial collateral ligament (MCL) injury; stars: bone marrow oedema.

ALL injury was defined as loss of clear continuity, irregularity or thinning of the ligament fibres, or abnormal signal intensity. MRI scans of insufficient quality or with non‐visualisation of the ALL were excluded from analysis. Medial and lateral anterior tibial translation were measured according to Harmes et al. [14] (Figure 3), a standardised method validated in ACL‐injured knees. Negative values indicate a relative posterior tibial position with respect to the femoral condyle.

**Figure 3:**
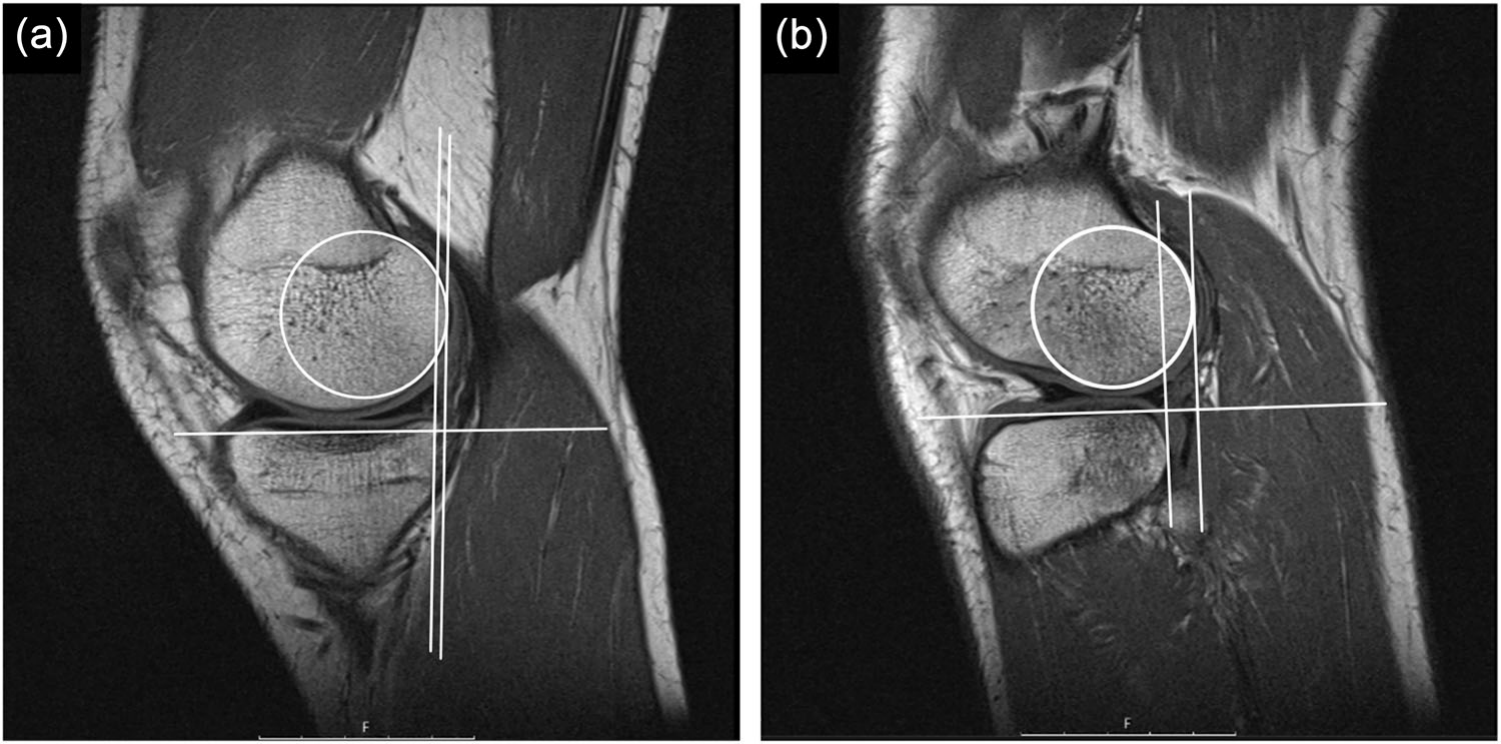
Measurement of medial and lateral anterior tibial translation according to the method described by Harmes et al. [14]. (a) For medial anterior tibial translation, the reference image is selected at the level of the femoral insertion of the medial gastrocnemius tendon—sagittal T1‐weighted magnetic resonance imaging (MRI). (b) For lateral anterior tibial translation, the reference image is identified at the level of the most medial aspect of the fibular head within the proximal tibiofibular joint – sagittal T1‐weighted MRI. In both measurements, a best‐fit circle is placed over the subchondral surface of the posterior femoral condyle. A line is drawn along the tibial plateau from its most anterior to its most posterior margin. A second line, perpendicular to the tibial plateau, is positioned tangentially to the posterior edge of the best‐fit circle, and a third line tangentially to the posterior tibial cortex. The distance between these two parallel lines defines the degree of static anterior tibial translation.

Bone bruise was defined as increased signal intensity on T2‐weighted images of the lateral femoral condyle or lateral tibial plateau. Medial and lateral meniscal injuries were defined as increased signal intensity, morphological disruption, or discontinuity of the meniscal tissue on proton density‐weighted sequences. MCL, LCL and PCL injuries were identified by the presence of ligament discontinuity, increased intrasubstance signal density, thickening (> 7 mm AP diameter for PCL), or surrounding oedema/fluid signal. LPTS and MBA were measured on the same sagittal slice according to Hudek et al. [23] and Sturnick et al. [52], respectively (Figure 4).

**Figure 4:**
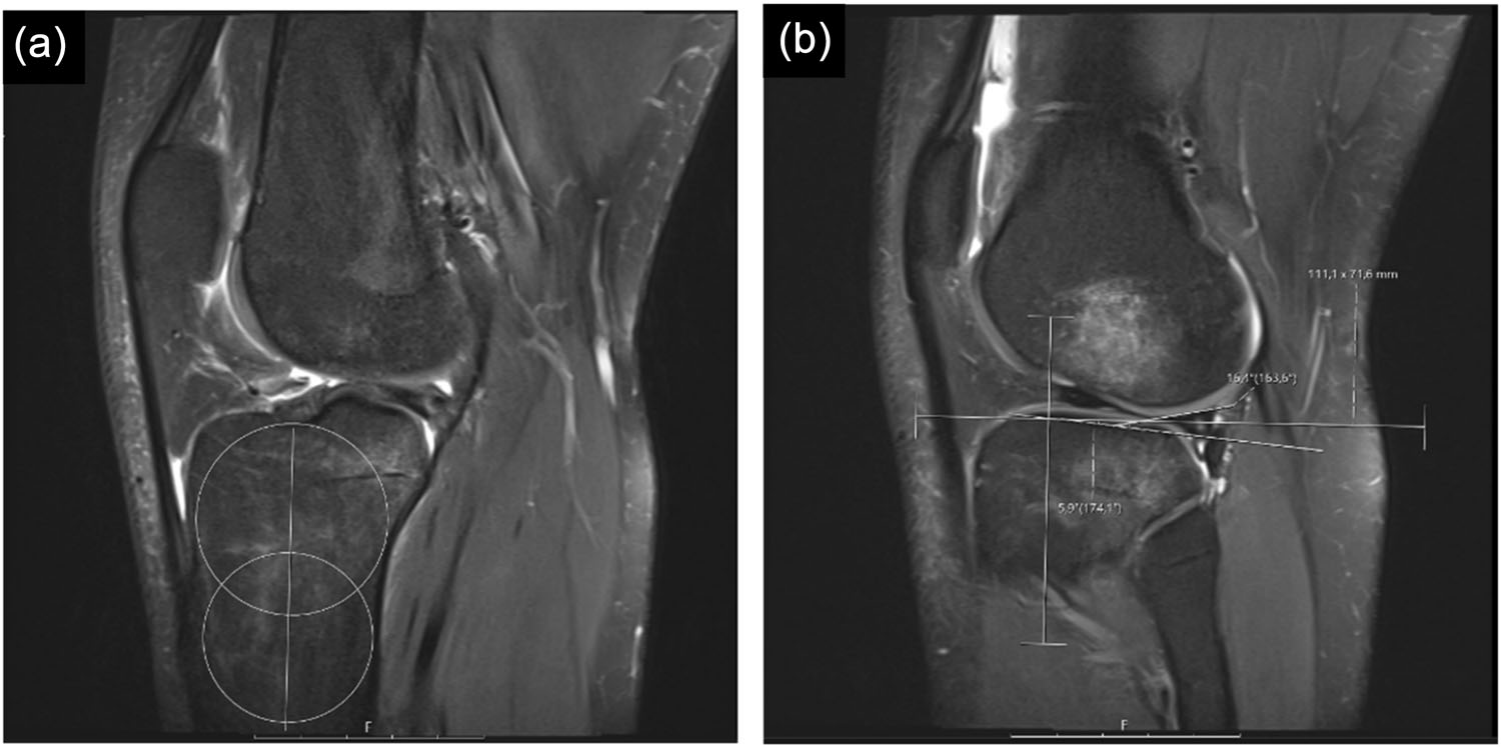
Measurement of lateral posterior tibial slope (LPTS) and meniscal bone angle (MBA) according to Hudek et al. [23] and Sturnick et al. [52], respectively. Sagittal proton density‐weighted magnetic resonance imaging (MRI) with fat saturation. (a) Identification of the longitudinal tibial axis using two best‐fit circles. (b) Measurement of LPTS and MBA on the sagittal slice of the lateral tibial plateau.

All angular measurements were reported to one decimal place, consistent with the demonstrated accuracy of MRI‐based angular measurement of 0.1° [9, 57].

### Statistical analysis

The sample size was calculated using G*Power 3.1.9.7 software. A small‐to‐moderate effect size (Cohen's *w *= 0.30) was assumed, with statistical power set at 80%, and a two‐sided α level of 0.05. Under these assumptions, the minimum required sample size was 88 patients. A post hoc power analysis for the primary outcome, if needed, was planned according to the observed effect size, group sizes, and significance level.

Statistical analysis was conducted using IBM SPSS Statistics for Windows, Version 30.0 (IBM Corp., Armonk, N.Y., USA). A *p*‐value of 0.05 was set for significance. Missing data were excluded from the analysis. As the distributions of continuous variables were non‐normal, nonparametric tests were used. Differences between groups were analysed using the Mann–Whitney *U* test, and data are presented as median and interquartile range (IQR). The Pearson *χ* ^2^ test and Fisher's exact test were used to compare the distribution of binary or categorical variables. The Wilcoxon signed‐rank test was used to compare non‐parametric continuous variables. Confidence intervals (CI) and effect sizes and were calculated to complement statistical significance testing [10]. Subgroup analyses were performed in patients who underwent MRI within 30, 60 and 90 days post‐injury, and p‐values were adjusted using the Bonferroni method to account for multiple comparisons. Multivariable logistic regression was performed to identify independent factors associated with ALL injury in patients with ACL injury.

## RESULTS

A total of 329 patients who underwent primary ACL surgery between January 2012 and December 2022 were assessed for eligibility. Once the exclusion criteria were applied (Figure 5), a total of 176 patients were included: 40 (22.7%) in the ALL injury group, and 136 (77.3%) in the control group (Figure 5). There was substantial interobserver agreement for the identification of anterolateral lesions (*κ* = 0.715, 95% CI [0.595–0.835]; *p* < 0.001), and the interobserver agreement was 89.2% (157 of 176 cases).

**Figure 5:**
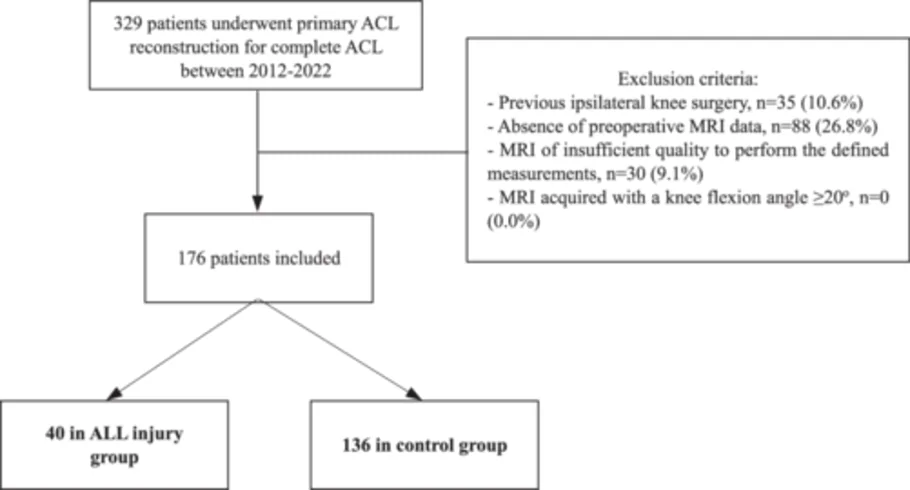
Patient selection flowchart (exclusion criteria were applied sequentially and mutually exclusively; each patient was counted only once, according to the first applicable criterion). ACL, anterior cruciate ligament; ALL, anterolateral ligament; MRI, magnetic resonance imaging.

Baseline demographic and clinical characteristics are presented in Table 1. Time from injury to MRI was also shorter in patients with ALL injury than in those with an intact ALL (41, IQR [26–140] vs. 91, IQR [41–233]; *p* = 0.029, *r* = −0.165). However, no differences in time to MRI were observed when the variable was analysed by month. There were no other significant differences in patients' baseline characteristics between the intact and injured ALL groups.

**Table 1:** Characteristics of study population.

Data	Control group (*n* = 136)^a^	ALL injury group (*n* = 40)^a^	*p*‐value^b^
Age (years)	26 [19–32]	26 [21–34]	0.483
Sex			0.667
Female	28 (20.6%)	7 (17.5%)	
Male	108 (79.4%)	33 (82.5%)	
Height (cm)	175 [169–179]	175 [170–182]	0.470
Unknown	10	2	
Weight (kg)	74 [65–85]	80 [68–92]	0.134
Unknown	3	3	
BMI (kg/m^2^)	24.9 [22.8–27.7]	25.8 [23.4–27.9]	0.231
Unknown	10	3	
Injured knee			0.030*
Right	84 (61.8%)	17 (42.5%)	
Left	52 (38.2%)	23 (57.5%)	
Injury mechanism			0.686
Traumatic contact	27 (19.9%)	8 (20.0%)	
Non‐traumatic	98 (72.1%)	27 (67.5%)	
Unknown	11 (8.1%)	5 (12.5%)	
Time to MRI (days)	91 [41–233]	41 [26–140]	0.029*
Time to MRI			0.237
<30 days	22 (64.7%)	12 (35.4%)	
30–60 days	21 (80.8%)	5 (19.2%)	
60–90 days	10 (76.9.7%)	3 (23.1%)	
>90 days	53 (82.8%)	11 (17.2%)	

Abbreviations: ALL, anterolateral ligament; BMI, body mass index; cm, centimetres; kg, kilograms; MRI, magnetic resonance imaging.

^a^ Median [interquartile range]; *n* (%).

^b^ Mann–Whitney *U* test for continuous variables; Pearson's chi‐squared test or Fisher's exact test for categorical variables.

* Statistically significant difference between groups (*p* < 0.05).

MRI anterolateral rotatory instability signs according to ALL status are summarised in Table 2. The prevalence of DLFNS (> 2 mm) was similar between patients with and without ALL injury (10/40 (25.0%) vs. 29/136 (21.3%); *χ* ^2^ = 0.242; *p* = 0.623; ϕ = 0.038). ALL injury was present in 10/40 (25.0%) of the DLFNS group and 29/136 (21.3%) of the non‐DLFNS group (absolute risk difference −3.7%, 95% CI [−18.8; 11.4]). Sensitivity analysis identified a minimum detectable effect size of *w*=0.211 at 80% power, whereas the observed effect corresponded to a post hoc power of 7.8%. Medial and lateral anterior tibial translation were also similar between the two groups. Analysis of the entire sample revealed significantly greater lateral than medial anterior tibial subluxation (medial 1.2, IQR [−1.3 to 3.2] vs. lateral 6.7, IQR [3.8–9.8]; *p* < 0.01; *r*_*s*_ = 0.405), suggesting increased tibial internal rotation, detailed in Table 3.

**Table 2:** Comparison of MRI anterolateral rotatory instability signs (DLFNS, medial and lateral anterior tibial translation) between control and ALL injury groups.

Data	Control group (*n* = 136)^a^	ALL injury group (*n* = 40)^a^	95% CI^b^	*p*‐value^c^	Effects size
DLFNS	29 (21.3%)	10 (25.0%)	[0.5–2.8]	0.623	0.038
LFNS depth (mm)	1.5 [1.1–1.8]	1.5 [1.1–2.0]	[−0.5 to 0.2]	0.705	−0.029
Medial anterior translation (mm)	1.2 [−1.2 to 3.2]	1.2 [−1.5 to 3.1]	[−0.4 to 0.5]	0.659	−0.033
Lateral anterior translation (mm)	6.8 [3.8–10.0]	5.4 [3.8–8.3]	[−0.2 to 0.6]	0.251	−0.087

Abbreviations: ALL, anterolateral ligament; CI, confidence interval; DLFNS, deep lateral femoral notch sign; LFNS, lateral femoral notch sign; mm, millimetres.

^a^ Median [interquartile range]; *n* (%).

^b^ Difference between means for continuous variables; odds ratio for categorical variables.

^c^ Mann–Whitney *U* test for continuous variables; Pearson's chi‐squared test or Fisher's exact test for categorical variables.

* Statistically significant difference between groups (*p* < 0.05).

**Table 3:** Comparison of medial and lateral anterior tibial translation within the ACL injury group (*n* = 176).

Data	ACL injury group (*n* = 176)		95% CI^b^	*p*‐value^c^	Effects size
	Medial (mm)^a^	Lateral (mm)^a^			
Anterior Translation (mm | )	1.2 [−1.3 to 3.2]	6.7 [3.8–9.8]	[4.9–6.2]	<0.001*	0.852

Abbreviations: ACL, anterior cruciate ligament; CI, confidence interval; mm, millimetres;

^a^ Median [interquartile range].

^b^ Odds ratio for categorical variables.

^c^ Wilcoxon signed‐rank test.

* Statistically significant difference between groups (*p* < 0.05).

MRI concomitant lesions and knee morphological characteristics are detailed in Table 4. The prevalence of bone lesions, medial and lateral meniscal tears, and injuries of the PCL, MCL, or LCL were not significantly different between groups (*p* > 0.05). Similarly, no statistically significant differences were observed in LPTS or MBA between patients with and without ALL injury.

**Table 4:** Comparison of MRI concomitant lesions and knee morphological characteristics between control and ALL injury groups.

Data	Control group (*n* = 136)^a^	ALL injury group (*n* = 40)^a^	*p*‐value^b^
Bone bruise	72 (52.9%)	24 (60.0%)	0.431
Medial meniscus	89 (65.4%)	22 (55.0%)	0.229
Lateral meniscus	69 (50.7%)	23 (57.5%)	0.431
MCL	36 (26.5%)	16 (40.0%)	0.099
LCL	8 (5.9%)	5 (12.5%)	0.160
PCL	2 (1.5%)	1 (2.5%)	0.541
LPTS (degrees)	4.7 [2.7–7.1]	3.5 [1.7–7.4]	0.299
MBA (degrees)	20.7 [16.3–24.2]	19.7 [16.3–23.9]	0.470

Abbreviations: ALL, anterolateral ligament; LCL, lateral collateral ligament; LPTS, lateral posterior tibial slope; MBA, meniscal bone angle; MCL, medial collateral ligament; MRI, magnetic resonance imaging; PCL, posterior cruciate ligament.

^a^ Median [interquartile range]; *n* (%).

^b^ Mann–Whitney *U* test for continuous variables; Pearson's chi‐squared test or Fisher's exact test for categorical variables.

According to previous results, the groups differed in time to MRI; therefore, subgroup analyses were performed in patients who underwent MRI within 30, 60 and 90 days post‐injury. In the <30‐day post‐injury subgroup (Table 5), patients with ALL injury showed a higher prevalence of DLFNS than those with intact ALL (7/12 (58%) vs. 6/22 (27.3%)). However, this difference did not reach statistical significance (*p* = 0.225). In the <60‐and <90‐day post‐injury subgroups, the differences were also not statistically significant (*p* = 0.468 and *p* = 0.579, respectively).

**Table 5:** Comparison of DLFNS presence between control and ALL injury groups in patients with time to MRI < 30 days.

Data	Control group (*n* = 22)^a^	ALL injury group (*n* = 12)^a^	95% CI^b^	*p*‐value^c^	Effects size
DLFNS (> 2 mm)			[0.8–16.4]	0.225	0.305
Present	6 (27.3%)	7 (58.3%)			
Absent	16 (72.7%)	5 (41.7%)			

Abbreviations: ALL, anterolateral ligament; CI, confidence interval; DLFNS, deep lateral femoral notch sign; mm, millimetres; MRI, magnetic resonance imaging.

^a^ Median [interquartile range]; *n* (%).

^b^ Odds ratio for categorical variables.

^c^ Fisher's exact test (*p*‐value adjusted using Bonferroni method).

Table 6 presents the results of the multivariable logistic regression analysis identifying variables independently associated with ALL injury in ACL‐injured patients. The presence of bone bruise was identified as the only independent predictor of ALL injury (adjusted odds ratio (OR) 2.192, 95% CI [1.012–4.748]; *p* = 0.047), suggesting that ALL injury may be part of a broader high‐energy trauma pattern. Conversely, LFNS depth and time to MRI were not independently associated with ALL injury after multivariable adjustment.

**Table 6:** Multivariable logistic regression analysis to identify independent factors associated with ALL injury in patients with ACL injury.

Data	Adjusted OR	95% CI	*p*‐value
BMI	1.024	[0.937–1.120]	0.598
Time to MRI	1.000	[0.999–1.000]	0.256
LFNS (mm)	0.826	[0.486–1.406]	0.482
Bone bruise			0.047*
Intact	—	—	
Injured	2.192	[1.012–4.748]	
LPTS (degrees)	0.996	[0.888–1.117]	0.944
Lateral meniscus			0.577
Intact	—	—	
Injured	0.809	[0.385–1.701]	

Abbreviations: ACL, anterior cruciate ligament; ALL, anterolateral ligament; CI, confidence interval; LFNS, lateral femoral notch sign; LPTS, lateral posterior tibial slope; mm, millimetres; OR, odds ratio.

* Statistically significant difference between groups (*p* < 0.05).

## DISCUSSION

In this MRI‐based study of 176 patients with complete ACL injury, concomitant ALL injury was identified in 40/176 (22.7%) of cases (interobserver agreement 89.2%, *κ* = 0.715), and concomitant bone bruise was the only independent factor associated with ALL injury (OR 2.192, 95% CI [1.012–4.748]). No significant association was observed between ALL injury and MRI anterolateral rotatory instability signs.

The only baseline difference between groups was a higher proportion of left‐sided injuries in the ALL injury group (23/40 (57.5%) vs. 52/136 (38.2%); *p* = 0.030). This finding is likely attributable to chance, as no established anatomical or biomechanical mechanism links knee laterality to ALL injury susceptibility. Several authors have proposed the DLFNS as an indirect marker of a more severe pivoting trauma pattern and a potential surrogate for ALC injury [38]. Dimitriou et al. reported that a deeper LFNS, measured on plain radiographs, was strongly associated with concomitant ALL injury in ACL‐injured knees and suggested a threshold of approximately 1.8 mm as a useful diagnostic cut‐off [7]. Moreover, LFNS greater than 2 mm has been linked to a steeper posterior tibial slope [4], a higher prevalence of lateral meniscal lesions [3, 4, 19, 29], and broader patterns of osseous and soft‐tissue injury [25, 29], reinforcing the concept of DLFNS as a radiographic marker of high‐energy pivot‐shift mechanisms rather than a marker of isolated ACL injuries. In contrast, our data did not demonstrate a significant association between DLFNS presence and ALL injury in the overall cohort control group.

Overall, ALL injury was identified in 40/176 (22.7%) of patients with ACL injury, which is consistent with the range reported in recent MRI‐based studies (11%–79%) [1, 34, 54]. These discrepancies may reflect differences in imaging protocols [15, 43], timing of MRI acquisition [43], thresholds used to define a “positive” DLFNS, and criteria for diagnosing ALL injury. DLFNS reflects the energy and kinematics of the initial injury event, it may not reliably indicate the specific pattern or extent of ALC injury [22, 27]. In the present study, neither medial nor lateral anterior tibial translation differed significantly between patients with and without ALL injury. In fact, static MRI‐based measurements of anterior tibial subluxation may not adequately capture the functional consequences of ALL disruption. Rotatory knee instability is a complex, dynamic phenomenon influenced by multiple anatomical structures, including the ACL, the ALC, and the Kaplan fibres [21, 42], which have been increasingly recognised as important contributors to rotational stability [28, 32, 53, 56]. Static supine MRI measurements may therefore underestimate subtle differences in rotational laxity that are more accurately detected through dynamic clinical tests, such as the pivot‐shift, or instrumented intraoperative assessment [8, 33]. Moreover, anterior subluxation primarily reflects sagittal‐plane instability, whereas the ALL and related anterolateral structures are believed to play a more prominent role in controlling axial rotation [41]. However, analysis of the entire sample, in ACL injured patients, revealed a statistically significant difference between medial (1.2, IQR [−1.3 to 3.2]) and lateral (6.7, IQR [3.8–9.8]) anterior tibial subluxation (*p* < 0.01; *r*_*s*_ = 0.405). This finding is clinically meaningful, as greater lateral than medial anterior tibial subluxation indicates increased tibial internal rotation. The observed median lateral anterior tibial subluxation of 6.7 mm exceeds the threshold of ≥ 6 mm previously associated with high‐grade rotatory instability in acute ACL injuries [35], and the lateral‐medial difference of approximately 5.5 mm further supports the presence of tibial internal rotation, consistent with previous reports demonstrating that compartmental asymmetry of anterior tibial subluxation serves as a proxy for tibial internal rotation in ACL‐injured knees [59]. These findings align with evidence that lateral compartment subluxation, rather than medial, is the primary MRI correlate of anterolateral rotatory instability [33].

The significant difference in time to MRI between groups (41, IQR [26–140] vs. 91, IQR [41–233]; *p* = 0.029; *r* = −0.165) observed in this study should be interpreted with caution, as MRI timing may influence the identification of osseous and soft‐tissue injury patterns [16, 17]. In the <30‐day post‐injury subgroup, patients with ALL injury showed a higher prevalence of DLFNS than those with intact ALL (7/12 (58.3%) vs. 6/22 (27.3%)), this difference did not reach statistical significance (*p* = 0.225). Moreover, time to MRI did not differ significantly when categorised by month (< 30, 30–60, 60–90, and 90 days; *p* = 0.237) and was not independently associated with ALL injury in the multivariable logistic regression analysis (OR 1.000, 95% CI [0.999–1.000]; *p* = 0.256). In the acute phase following ACL injury, bone marrow oedema and impaction injuries are more conspicuous, potentially increasing DLFNS sensitivity [37]. Similarly, ALL visualisation may be more reliable early post‐injury, before scar formation, tissue remodelling, or partial healing [16, 43]. In fact, the diagnostic performance of MRI for ALL injury varies widely in the literature, with reported prevalence ranging from 11% to 79% in ACL‐injured knees and interobserver agreement ranging from near‐chance (*κ* = 0.04) to near‐perfect (*κ* = 1.0), depending on field strength, imaging protocols, and definition criteria [1, 34, 54]. This variability may partly reflect differences in MRI acquisition timing, as studies performing imaging closer to the time of injury consistently report higher rates of ALL injury detection. In the present study, the substantial interobserver agreement (89.2%; *κ* = 0.715) supports the reproducibility of ALL injury assessment and reinforces confidence in the accuracy of our MRI‐based evaluation. Among studies reporting MRI acquisition within approximately 2 weeks of injury, ALL injury prevalence ranged from 30% to 94% [1, 47, 48], whereas studies with longer imaging intervals reported substantially lower rates, as low as 10.7% [1]. Notably, a recommendation to perform MRI within 6 weeks of injury to optimise ALL visualisation has been previously proposed [30]. The lower ALL injury prevalence observed in the present cohort (40/176 (22.7%)) is consistent with the relatively longer time to MRI in a substantial proportion of patients, which may have contributed to underestimation of ALL injury through tissue remodelling and partial healing. These findings highlight the substantial methodological heterogeneity that limits cross‐study comparisons and may partly account for the discrepancies observed between the present and previous reports [18]. Delayed imaging may thus underestimate subtle anterolateral lesions [16], while early imaging better captures the acute injury pattern [17, 58]. These temporal patterns may also reflect the biological course of ALL healing: fibrous scar tissue formed during ligament remodelling can exhibit MRI signal intensity approaching that of an intact ligament, potentially leading to false negatives. Although our data did not demonstrate a statistically significant relationship between time to MRI and ALL detection, ALL is likely more readily visualised in the acute phase, before scar‐related changes develop. Accordingly, MRI should ideally be performed within 6 weeks of injury to optimise visualisation of the ALL [30]. These temporal differences should ideally be performed when interpreting DLFNS‐ALL associations and comparing study findings, particularly in the context of ALC contributions to rotatory knee stability.

These findings were further reinforced by the multivariable logistic regression analysis, which showed that the presence of bone bruise was the only variable independently associated with ALL injury (adjusted OR 2.192, 95% CI [1.012–4.748]; *p* = 0.047), with no association observed between ALL injury and time to MRI or LFNS. The association with concomitant bone bruise may be consistent with a broader injury pattern in patients with ALL injury, rather than an isolated finding. Accordingly, these findings do not support the use of DLFNS as a reliable, isolated imaging marker for concomitant ALL injury in ACL‐injured knees, contrasting with previous reports by Dimitriou et al. [7]. This discrepancy should not be interpreted as invalidating the findings of Dimitriou et al. [7], but rather as reflecting differences between study populations and methodologies, notably, the use of plain radiographs to assess the lateral femoral notch in their study, compared with MRI in the present cohort, as previously discussed. Although DLFNS has been associated with high‐energy pivoting mechanisms and broader injury patterns [19, 29, 38], our data do not demonstrate a direct relationship between its presence and ALL injury on MRI. Similarly, the absence of significant differences in anterior tibial translation reinforces that static supine MRI measurements may not adequately reflect the complex biomechanics of rotatory knee instability, as the ALC likely contributes to rotational control in ways not fully captured by sagittal‐plane subluxation assessments [11, 56]. From a clinical perspective, the isolated use of DLFNS to guide decisions regarding anterolateral augmentation is not supported by the present data [27, 49], and comprehensive assessment incorporating injury mechanism, rotational laxity, and patient‐specific risk factors appears warranted [49]. Overall, our findings are comparable with the literature, suggesting that DLFNS may primarily influence injury severity and pivoting mechanism, rather than serving as a specific surrogate for ALL involvement.

The current study has several strengths. Primarily, the sample size of 176 ACL‐injured patients exceeded the a priori power calculation (*n* = 88), ensuring adequate power to detect meaningful differences. Data were derived from a large institutional database over a 10‐year period using consecutive patient inclusion, contributing to a more representative and consistent study cohort. Furthermore, the primary exposure variable (ALL injury) was independently assessed by two reviewers, with discrepancies resolved by a senior musculoskeletal radiologist, thereby ensuring high internal validity and reducing the interobserver variability inherent to the identification of this structure. There was substantial agreement in the assessment of ALL injury (*κ* = 0.715, 95% CI [0.595–0.835]), supporting the validity of the classification used in this study.

Several limitations must, however, be acknowledged. First, the cross‐sectional design inherently limits causal inference regarding the temporal relationships between the different imaging findings. Second, static measurements may not fully capture the dynamic rotatory instability assessed with pivot‐shift testing, once the diagnosis of ALL injury relied on MRI findings without dynamic clinical assessment or surgical correlation. Third, MRI was performed using a 1.5 T system, which may reduce sensitivity for subtle ALL injuries compared to 3.0 T higher‐field systems. Additional limitations include potential selection bias due to the single‐centre study design and inclusion of surgically treated ACL injuries only, which may limit generalisability to other clinical settings or non‐surgical populations. Additionally, the exclusion of a substantial proportion of the initially eligible cohort (*n* = 118 of 329, 35.9%) may have introduced selection bias, as patients without preoperative MRI (*n* = 88) or with insufficient image quality (*n* = 30) may have differed systematically from those included. Nevertheless, these exclusions were considered unlikely to be related to ALL status, and thus the included sample likely remained representative of the study population. The post hoc analysis revealed limited sensitivity to detect small associations (post hoc power of 7.8%), suggesting that the primary findings should be interpreted with caution. Accordingly, this study cannot exclude small between‐group differences. Although the achieved sample size exceeded the a priori estimate, these results provide a basis for future prospective studies with larger sample sizes. Moreover, tibial translation was measured using a recently published method [14] that has limited external validation to date; however, the reproducibility of this method in retrospective MRI datasets requires further confirmation. Finally, there was a wide variation in time from injury to MRI across the study cohort. The overall sample exceeded the a priori calculation, yet no significant differences in ALL prevalence were observed when time to MRI was analysed by month, nor was time to MRI independently associated with ALL injury in logistic regression. The relatively small subgroup sizes, particularly in the <30‐day MRI group, may have reduced statistical power and underestimated this association. These findings highlight the need for prospective studies with standardised early imaging protocols.

## CONCLUSIONS

In this study, no direct association was found between ALL injury and MRI signs of anterolateral rotatory instability. Concomitant bone bruise was the only independent factor associated with ALL injury. This finding may be consistent with ALL injury occurring within a broader injury pattern, which may not be reliably captured by DLFNS alone. The significantly greater lateral than medial anterior tibial subluxation observed across the cohort suggests increased tibial internal rotation in ACL‐injured knees, independent of ALL injury status. Further prospective studies integrating dynamic evaluation of rotational instability are warranted to clarify the relationship between anterolateral structures and imaging‐based markers in ACL‐injured knees.

## AUTHOR CONTRIBUTIONS

All authors contributed to the study conception and design. Material preparation and data collection were performed by David Santos‐Nunes, Daniel Francisco Medina‐Dias, Pedro Diogo Santos Poças, Pedro Miguel Ferreira Sá and Miguel Henriques Castro. Analysis was performed by Daniel Francisco Medina‐Dias. The first draft of the manuscript was written by David Santos‐Nunes and Daniel Francisco Medina‐Dias, and all authors commented on previous versions of the manuscript. All authors read and approved the final manuscript.

## FUNDING INFORMATION

The authors have no funding to report.

## CONFLICT OF INTEREST STATEMENT

The authors declare no conflicts of interest.

## ETHICS STATEMENT

This study was approved by the Ethics Committee of ULS São João (CE‐425‐2025). Informed consent was waived by the Ethics Committee due to the retrospective nature of the study.

## Data Availability

Due to privacy reasons, the data supporting the findings of this study can be obtained from the corresponding author upon reasonable request.
